# Clinical utility and impact of autopsies on clinical practice among doctors in a large teaching hospital in Ghana

**DOI:** 10.3402/gha.v7.23132

**Published:** 2014-02-03

**Authors:** Edem Tette, Alfred E. Yawson, Yao Tettey

**Affiliations:** 1 Department of Community Health, College of Health Sciences, University of Ghana Medical School, Korle-Bu, Accra, Ghana; 2 Department of Pathology, College of Health Sciences, University of Ghana Medical School, Korle-Bu, Accra, Ghana; 3 Office of the Provost, College of Health Sciences, Korle-Bu, Accra, Ghana

**Keywords:** autopsy, clinical utility, clinicians, teaching hospital, Ghana

## Abstract

**Background:**

Autopsies can provide a good indication of the quality of patient care, in terms of the accuracy of clinical diagnosis and the quality of treatment given.

**Designs:**

This was a cross-sectional study among clinicians at the Korle-Bu Teaching Hospital (KBTH) in 2012. Data were collected with a 69-item, self-administered, structured questionnaire. A total of 215 questionnaires were sent out and 119 clinicians responded. Data were collected on the benefits and utility of autopsies for medical practice, care of patients, and management of clinical wards. Survey data were analyzed by simple descriptive statistics (i.e. proportions, ratios, and percentages). Data were analyzed using SPSS version 21.

**Objective:**

This study examined the views of clinicians regarding the utility of autopsies and their influence on clinical practice in a large teaching hospital in Ghana.

**Results:**

Overall, clinicians in KBTH agreed that autopsy reports are useful in answering clinical questions (55/119; 46.2%), confirming or verifying clinical diagnoses (54/119; 45.4%), providing information on unsuspected diagnoses (40/119; 33.6%), and for medical education (90/119; 75.6%). Overall, 70/119 (58.8%) of clinicians agreed that autopsy findings improve completeness and reliability of death certification and provide information on clinical effectiveness of treatment and patient management. However, only 23/119 (19.3%) of sampled clinicians had personal interactions with a pathologist during autopsy processes and 93/119 (78.2%) had not attended any autopsy demonstrations in the past 6 months. Attendance of pathologists at clinicopathological meetings of clinical departments of KBTH was minimal. Unfortunately, the use of autopsy reports for auditing clinical diagnostic performance was not seen as essential.

**Conclusion:**

Strengthening the interaction between doctors and pathologists is essential in improving the autopsy process and utilization in the hospital. KBTH should create opportunities for doctors to attend autopsy demonstrations and for pathologists to attend clinicopathological meetings in the hospital.

Autopsy rates have declined gradually over several decades in many parts of the world (1–3). Autopsy examinations can provide a good index of the quality of patient care, both in terms of the accuracy of clinical diagnosis and the quality of treatment given (4–6). Despite the well-established role of autopsy in disclosing clinical diagnostic inaccuracy among clinicians (7–10), many factors underlie the decline in undertaking autopsies.

The reasons for the decline are many, varied, and complex. Clinicians have tended to undervalue the clinical importance of autopsy and have become wary of requesting an examination that may reveal discrepancies between diagnosis and management (4, 7–12). Another potent reason is likely to be the attitudes of both health care professionals (negative attitudes and unwillingness to request autopsies) and that of the general public towards autopsies (due to cultural and religious beliefs) (5). In addition, the presence of modern diagnostic techniques, the fear of litigation from misdiagnosis and a general attitude among clinicians that autopsies are no longer relevant, may account for this decline (4, 5, 13).

The cost implications of an effective clinical pathology service are also significant. Limited numbers of trained pathologists and insufficient capacity to cover biopsy as well as autopsy workloads, especially in resource-limited settings, are important factors contributing to this decline (4, 6, 7).

In spite of the reduced rates of autopsies, rates of major errors in clinical diagnoses seem to have increased, and in some Western countries, despite the availability of sophisticated diagnostic techniques, there are observed discrepancies between clinical and autopsy diagnoses (4–8). In resource-limited settings with limited availability and application of sophisticated diagnostic techniques, the value and utility of autopsies are paramount. Autopsies, apart from being an important tool for improving clinical diagnostic accuracy, and for medical education and acquisition of new knowledge, are also a vital tool for medical quality assurance (14, 15). Some studies have also shown that by conducting autopsies and correlating the findings with clinical details, results can be used to develop effective diagnostic algorithms for improving clinical practice (16, 17).

The need for an efficient autopsy service and increased utilization of autopsy services by health practitioners to improve clinical care in a large teaching hospital, such as the Korle-Bu Teaching Hospital (KBTH), is of paramount importance. Overall, between 3,000 and 5,500 (average 4,100) autopsies are performed annually at the hospital, of which about 20% are hospital autopsies. The mean autopsy rates for the clinical departments of the hospital range from 30 to 60% (18). The aim of the study was to examine the views of clinicians regarding the utility of autopsies, its influence on clinical practice, and how the autopsy service can be improved at KBTH.

## Methods

This was a cross-sectional study of doctors at KBTH during 2012. The data were collected using a 69-item, self-administered, structured questionnaire. A total of 215 questionnaires were sent out and 119 doctors responded.

### Site of study

KBTH, a tertiary health care facility in Ghana, was the survey site. KBTH has a bed capacity of 2,000 and over 3,000 staff (18). In 2010, 29,757 clients were seen on average per month, average daily outpatient attendance was 1,500, and average daily admissions was 150 (18). The autopsy service in KBTH is provided by the Department of Pathology. The Department of Pathology in the University of Ghana Medical School (UGMS) provides surgical pathology, cytology/cytopathology, and autopsy services. It is involved in undergraduate, postgraduate, and residency training and research in surgical pathology, cytopathology, and autopsy.

The Pathology department currently has six consultant pathologists, six specialist pathologists, and five residents in training as well as some biomedical scientists and administrative staff. The department manages and operates the mortuary unit, which has a storage capacity of 350 bodies and stores between 8,500 and 10,000 bodies annually. The mortuary serves KBTH and the general public (18).

Overall, between 3,000 and 5,500 (average 4,100) autopsies are performed annually and about 20% of these are hospital autopsies. In 2010, the autopsy rates at KBTH were as follows: Department of Child Health, 30%; Department of Obstetrics and Gynaecology (OBGYN), 30%; Department of Surgery, 38%; and Department of Medicine, 60% (18). In KBTH, autopsies are requested by attending physicians, without seeking consent from families.

### Sampling methods/selection of survey sites

Doctors from the main clinical departments, that is, Internal Medicine, Surgery and Allied Surgery, and Child Health and OBGYN were involved in the survey. These service centers were selected based on the clinical services provided by the doctors. The survey targeted all doctors in these departments, and questionnaires were distributed in each department through the heads of department.

### Study population

The population for the survey included doctors from all areas of the hospital. These included house officers, medical officers, senior medical officer/residents, senior residents, and consultants.

### Data collection

The data were collected using a 69-item, self-administered, structured questionnaire. The questionnaire solicited information on the background of the respondent, including sex, current status, and department of work, as well as the main reasons for autopsy requests. Background data also included knowledge on laws and legislation concerning autopsies in Ghana, whether the respondents attended departmental clinicopathological or mortality meetings, and whether KBTH pathologists were involved in these mortality meetings. Data on the benefits and utility of autopsy to medical practice, care of patients, management of the clinical wards, and teaching of medical students in KBTH were also collected.

### Data analysis

Survey data were analyzed by simple descriptive statistics (i.e. proportions, ratios, and percentages) and summarized in tables. Data were entered into Microsoft Excel 2007 and imported into SPSS version 21, and analyzed.

### Ethical issues

Ethical approval for the survey was obtained from the UGMS Ethical and Protocol Review Committee (Protocol Identification Number: MSEt/M.11-P 5.8/2011–2012). Clearance was also received from the management of the KBTH and heads of clinical units where the survey was conducted.

## Results

A total of 215 questionnaires were sent out and 119 doctors responded, giving a response rate of 55.3%. There were more males (67; 56.3%) than females (52; 43.7%), with a male: female ratio of 1.3:1. The majority of the doctors involved in the survey were senior medical officers/residents (45; 37.8%). There were 19 consultants (16.0%) and 17 senior residents (14.3%), as indicated in Table 1.

**Table 1:** Status and sex distribution of 119 doctors of Korle-Bu Teaching Hospital involved in the survey

	Sex of respondent		
		
Current position	Male	Female	Total
House officer	18 (26.9%)	16 (30.8%)	34 (28.6%)
Medical officer	1 (1.5%)	3 (5.8%)	4 (3.4%)
Senior medical officer/resident	28 (41.8%)	17 (32.7%)	45 (37.8%)
Senior resident	10 (14.9%)	7 (13.5%)	17 (14.3%)
Consultant	10 (14.9%)	8 (17.3%)	19 (16.0%)
Total	67 (100%)	52 (100%)	119 (100%)

Among the doctors, the two most common reasons for requesting autopsies were to answer clinical questions (55; 46.2%) and in cases of uncertain diagnosis (54; 45.4%), as shown in Table 2. The other main reasons for autopsy requests as stated by the doctors were to confirm clinical diagnoses (40; 33.6%) and in cases meeting conditions for the coroner (39; 32.8%). Among all 119 doctors, 23 (19.3%) had personal interactions with pathologists during the process of autopsy, but the majority (93; 78.2%) had not attended any autopsy demonstrations in the past 6 months. However, almost all of the doctors (111; 93.3%) stated that they attend monthly mortality or clinicopathological meetings in their department/unit and a high number (90; 75.6%) also indicated that autopsy reports are discussed at such meetings. Only three respondents reported the attendance of pathologist at the monthly departmental clinico-pathological meetings.

**Table 2:** Basic information of 119 doctors of Korle-Bu Teaching Hospital regarding autopsies

Characteristic	Frequency	Percentage
Reasons for autopsy request		
Answer clinical questions	55	46.2
Uncertain diagnosis	54	45.4
Confirm clinical diagnosis	40	33.6
Coroner's case	39	32.8
Family request	2	1.7
Explain sudden questionable deaths	1	0.8
Personal interaction with pathologist during the process of autopsy		
Yes	23	19.3
Number of autopsy demonstrations attended by doctors in the past 6 months		
0	93	78.2
1	16	13.4
2	3	2.4
5 or more	7	5.9
Monthly mortality or clinicopathological meeting in department/unit		
Yes	111	93.3
Autopsy reports discussed at meetings		
Yes	115	96.8
Participation of pathologist or personnel from Department of Pathology in mortality meetings		
Yes	3	2.5

As indicated in Table 3, 78 (65.5%) of the doctors strongly agreed that autopsy was a tool to aid clinical assessment and 58 (48.7%) strongly agreed that autopsy was a good means of quality assurance and control of clinical work. Also, 97 (81.5%) doctors disagreed that autopsy was less relevant because of medical and technological advancement and a majority (67; 56.3%) reported that autopsy requests in the past 6 months resulted in previously unsuspected diagnoses, while 65 (54.6%) reported improved recognition of non-classical disease presentations. Other benefits of autopsies were that autopsy findings altered patient management for 51 doctors (42.9%), especially in identifying deep vein thrombosis, and for 20 doctors (16.8%) autopsy reports influenced concerns about drug efficacy; drugs mentioned included generic forms of ceftriaxone and co-amoxiclav, Clexane (subcutaneous heparin), and some antihypertensive drugs. In addition, 70 doctors (58.8%) agreed that autopsy reports influenced the issue of death certificates, especially the ability to state the exact cause of death (23; 19.3%), being more specific about cause of death (13; 10.9%) and in conditions where clinical cause of death was unknown (6; 5.0%).

**Table 3:** Stated utilities and benefits of autopsies among 119 doctors of Korle-Bu Teaching Hospital

Characteristic	Frequency	Percentage
Doctors consider autopsy a tool to aid clinical assessment process		
Strongly agree	78	65.5
Agree	41	34.5
Doctors consider autopsy a good means of quality assurance and control of clinical work		
Strongly agree	58	49.1
Agree	54	45.7
Uncertain	7	5.2
Doctors consider autopsy less relevant because of medical and technological advancement		
Agree	13	10.6
Uncertain	9	8.0
Disagree	97	81.4
Doctors’ autopsy request in the past 6 months resulted in further or unsuspected diagnosis		
Yes	67	56.3
Autopsy findings alter patient management by doctors		
Yes	51	42.9
Autopsy findings improved recognition of non-classical disease presentation		
Yes	65	56.0
Autopsy report influenced doctors’ issue of death certificate		
Yes	70	58.8
Doctors’ view on how autopsy report influenced the issue of a death certificate		
Able to state exact cause of death	23	19.3
Be more specific about cause of death	13	10.9
Cause of death is unknown	6	5.0
Additional diagnosis	3	2.5
Cause of death is known	3	2.5
Confirm clinical diagnosis	3	2.5
It provides closure to uncertain cases	1	0.8
Autopsy report influenced doctors’ concern about drug efficacy		
Yes	20	16.8

Other benefits of autopsies for doctors in KBTH, as shown in Table 4, are that autopsy reports influenced administrative decisions and guidelines on patient management on the ward and also influenced how patients were investigated. The doctors indicated that autopsy reports heightened clinical suspicion of conditions such as deep venous thrombosis or pulmonary edema, pulmonary and extrapulmonary TB, and some cancers, leukemias, and lymphomas. Other conditions reported are shown in Table 4.

**Table 4:** Other stated utilities and benefits of autopsies among 119 doctors in Korle-Bu Teaching Hospital

Characteristic	Frequency	Percentage
Autopsy report influenced administrative decisions on the ward		
Yes	26	21.8
Autopsy report influenced guidelines on patient management on the ward		
Yes	46	38.7
Autopsy report influenced investigation of patients on the ward		
Yes	42	35.3
Disease condition for which autopsy heightened doctors’ clinical suspicion (*N*=119)		
Deep vein thrombosis (DVT)/pulmonary embolism (PE)	9	7.6
Pulmonary TB/extrapulmonary TB	4	3.4
Cancers/leukemia/lymphoma	4	3.4
Pyogenic meningitis	3	2.5
Aortic aneurysm	3	2.5
Gastric perforation	2	1.7
Pneumonia	2	1.7
Typhoid perforation	2	1.7
Others (abdominal TB, acute chest syndrome, bronchoalveolar carcinoma, cerebral malaria, disseminated TB, etc.)	14	11.8
Autopsy report influenced infection control procedures on the ward		
Yes	15	12.6
Autopsy report influenced public health reporting obligations of doctors		
Yes	8	6.7
Autopsy report influenced the teaching of medical students (among doctors who teach)		
Yes	17	14.3
Autopsy report influenced risk management procedures on the ward		
Yes	18	15.1
Autopsy report influenced communication with families		
Yes	60	50.4
Doctors’ view on how autopsy reports influenced communication with families		
Able to explain cause of death better to families	22	18.5
Increased confidence in communication	6	5.0
Able to give a precise diagnosis	3	2.5
Ability to explain cause of death helps families to accept	2	1.7
Able to counsel them with the exact diagnosis	2	1.7
Others (autopsies bring out the need for proper patient education, promote better understanding among family members, improve the demand for autopsies, etc.)	11	9.2

Communication of doctors with families of patient was mentioned to have been improved by autopsy reports by 60 of the doctors (50.4%), especially regarding their ability to explain cause of death better to families (22; 18.5%), increased confidence of doctors in communicating with families (6; 5.0%), and the ability to give a precise diagnosis (3; 2.5%). Other benefits in communication with families garnered from autopsy reports are indicated in Table 4.

However, only a few doctors stated that autopsy reports influenced infection control procedures on the ward, public health reporting obligations of doctors, the teaching of medical students (among doctors who teach), and risk management procedures on the ward.

The views of doctors on the impact of autopsies in their clinical practice in the teaching hospital are shown in Table 5. In summary, doctors indicated that autopsies were useful in confirming or verifying clinical diagnosis (100; 84.0%), answering clinical questions (92; 77.3%), providing information on otherwise unsuspected diagnoses (91; 76.5%), and for medical education (90; 75.6%). Other important views on the utility of autopsies were that they improved the completeness and reliability of death certification (79; 66.4%), provided a basis for research among doctors (61; 51.3%), and provided information on the clinical effectiveness of treatment (37; 31.3%). Only one respondent mentioned the use of autopsy reports for auditing clinical diagnosis performance.

**Table 5:** Views of 119 doctors at Korle-Bu Teaching Hospital on the benefits of autopsies in their practice

Characteristic	Frequency	Percentage
Confirming or verifying clinical diagnosis	100	84.0
Answering clinical questions	92	77.3
Provide information on unsuspected diagnosis	91	76.5
Medical education	90	75.6
Improve the completeness and reliability of death certification	71	59.7
Research	61	51.3
Provide information on the clinical effectiveness of treatment	37	31.3
Auditing clinical diagnosis performance	1	0.8

## Discussion

Doctors from all categories in KBTH requested autopsies and there were more males than females. Residents in training were in the majority. These observations are not surprising in this teaching hospital where there are more male doctors, and the day-to-day care and management of patients is by these residents who work directly with the house officers (18). Increasing the frequency of autopsy requests among this category of doctors in training will enhance the utility of autopsies in the clinical management of patients.

The main reasons for requesting autopsies by doctors in KBTH were to answer clinical questions, in cases of uncertain diagnosis, to confirm clinical diagnoses, and in conditions for the coroner. These reasons are in conformity with other studies which indicated that autopsy examination provides a good index of the quality of patient care, in terms of the accuracy of clinical diagnosis and the quality of treatment given (4–7). Also, evidence exists that autopsies play an important role in disclosing clinical diagnostic inaccuracy among clinicians (7–10). In contrast, requesting autopsies in response to demand from family members was very low among the doctors. Potential explanations may likely be that doctors conform to protocols on requesting autopsies regardless of whether families request one or not. However, other studies have shown autopsy requests have been declined for multiple reasons (1–3), one of which is the fear of litigation by families who are increasingly becoming well informed on discrepancies between clinical and autopsy findings (4, 6, 8).

The utility of autopsies in the practice of doctors in KBTH was amply demonstrated by the survey. Over three-quarters of sampled doctors strongly agreed that autopsy was a tool to aid clinical assessment, and more than half of these indicated autopsy was a good means of quality assurance and control of clinical work. Post-mortem examination findings have been shown in other studies to be a vital tool for medical quality assurance (14, 15).

Despite advances in medical technology contributing to a global decline in the use of autopsies (1, 4, 6–8, 13), 97 (81.4%) of the responding doctors in this hospital maintained that autopsies are still relevant to clinical practice and 67 (56.3%) further stated that autopsy requests in the past 6 months had resulted in making an otherwise unsuspected diagnosis, which had improved recognition of non-classical disease presentations. Major errors of clinical diagnoses seem to have increased globally, and in some Western countries, despite the availability of sophisticated diagnostic techniques, there are observed discrepancies between clinical and autopsy diagnoses (4, 6–8). Making better use of autopsies in improving clinical practice among doctors practicing in resource-limited settings, with low rates of uptake and the application of advanced medical technology, is imperative.

Other benefits of autopsies mentioned in the survey included raising suspicion and improving management of some clinical conditions (e.g. deep vein thrombosis, pulmonary embolism, pulmonary and extrapulmonary TB, some cancers, leukemia, and lymphoma, aortic aneurysm, gastric perforation, and typhoid perforation). These conditions may pose diagnostic challenges, and autopsy findings may improve the skill and increase the index of suspicion for diagnosis among clinicians. Some studies have shown that conducting autopsies and correlating the findings with the clinical picture are a means for developing proper diagnostic algorithms to improve clinical practice (16, 17).

Death certification was also reported to have been improved as a result of autopsy reports by 70 (58.8%) of the sampled doctors, because autopsy reports state the exact cause of death, and are more specific about cause of death, especially when clinical cause of death is uncertain. In addition, doctors agreed that autopsy reports influence administrative decisions and guidelines on patient management on the wards, influence how patients are investigated and raise concerns about the efficacy of some medications (especially non-proprietary forms of some antibiotics).

Communication with patients and families continues to be a challenge for health professionals for a myriad of reasons (19, 20), including lack of time due to work overload and inadequate skills in communicating professionally. A good indicator for assessing quality of health care delivery is the proportion of patients who were told their diagnosis and who understood what the health worker said (21, 22).

An important utility of autopsies identified among 60 (50.4%) of the doctors was that communications with families of patients were improved by autopsy reports, especially regarding the ability to better explain cause of death to families, increased confidence of doctors in communicating with families, and the ability to give a precise diagnosis. In addition, post-mortem findings were said to bring out the need for proper patient and family education, promote a better understanding among family members on the cause of death of relations, and also improve the demand for more autopsies among doctors. These findings generally are in accordance with those from studies in other tertiary health care facilities (23, 24). The KBTH's patients’ rights and responsibilities document engenders the provision of essential information to clients in a professional manner (25); that autopsies do help in providing essential information on patients to families in a professional manner was one benefit of autopsies reported from the survey.

Despite the obvious utilities and benefits of autopsies, some concerns need to be mentioned. Only a quarter of the doctors had personal interaction with the pathologist during the process of autopsy and a majority (93; 78.2%) had not attended any autopsy demonstrations in the past 6 months. Attendance of pathologists at clinicopathological meetings of the clinical departments of the hospital was almost non-existent. Other concerns were that autopsy reports neither influenced infection control procedures on the ward, nor improved public health reporting obligations of doctors. Only one doctor reported the use of autopsy reports for auditing clinical diagnosis performance; obviously clinical auditing using autopsy reports needs to be encouraged in KBTH.

Interactions between requesting doctors and pathologists are essential in improving the autopsy process and utilization in a hospital (12, 14, 15, 23, 24). Interestingly, despite the relatively large numbers of clinicians recognizing the utility of autopsy in their clinical practice, there seemed to be almost no communication between clinicians and pathologists – pathologists do not go to mortality meetings, and clinicians do not go to autopsies. No matter how good autopsies are in principle, if the findings are not put into a clinical context and intimated to the clinicians, then they are worthless.

A potential policy measure will be for the Department of Pathology to assign residents in training to attend the monthly clinicopathological meetings of the Clinical Departments of the Hospital. KBTH should create opportunities for doctors to attend autopsy demonstrations probably as continuous professional development or as annual appraisal requirement. Clinicopathological meetings of the departments in the hospital should also include updates and presentations on utility of autopsies to clinical care of patients. A limitation of this analysis was the relatively low response rate of 55.3%. Doctors who did not respond might have provided other dimensions to the theme of the survey.

## Conclusions

Autopsies seemed to impact on the clinical care of patients, management of clinical wards, and the practice of clinicians in this large teaching hospital. Strengthening the interactions between requesting doctors and pathologists is essential in improving the autopsy process and utilization of autopsy findings in the hospital. The teaching hospital should create opportunities for doctors to attend autopsy demonstrations and include updates and presentations on the utility of autopsies during the clinicopathological meetings of the departments in the hospital.
